# Importance of Patient–Provider Communication to Adherence in Adolescents with Type 1 Diabetes

**DOI:** 10.3390/healthcare6020030

**Published:** 2018-03-28

**Authors:** Niral J. Patel, Karishma A. Datye, Sarah S. Jaser

**Affiliations:** Department of Pediatrics, Vanderbilt University Medical Center, Nashville, TN 37232, USA; niral.patel@vumc.org (N.J.P.); karishma.a.datye@vanderbilt.edu (K.A.D.)

**Keywords:** type 1 diabetes, adolescents, providers, self-management, patient-HCP communication, patient satisfaction

## Abstract

Effective communication between pediatric diabetes patients and their providers has the potential to enhance patient satisfaction and health outcomes, as well as improve diabetes-related self-management. In this review, we highlight the importance of communication between patients and providers, focusing on the effect of communication on adherence in the high-risk population of adolescents with type 1 diabetes. We synthesize the literature describing patient–provider communication in pediatric populations and provide implications for practice that focus on the most relevant, modifiable factors for improving self-management in adolescents with type 1 diabetes.

## 1. Introduction

**Figure 1:**
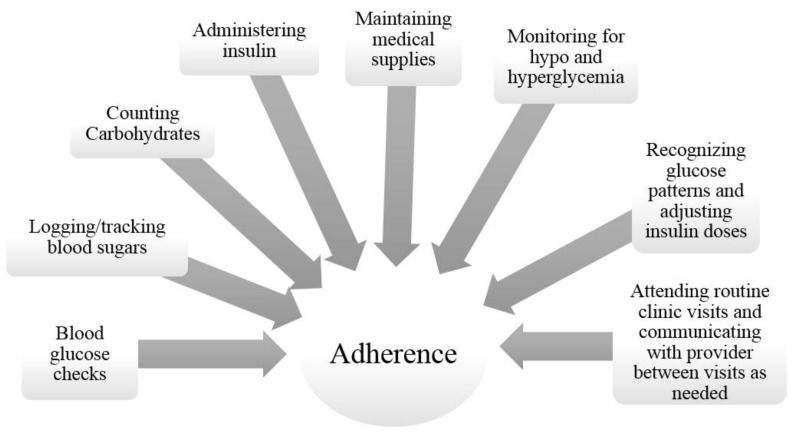
Type 1 Diabetes Treatment Regimen Tasks Associated with Adherence. Adapted from: Jaser et al. 2016 [4].

Most adolescent patients with type 1 diabetes (T1D) do not meet treatment goals, which increases their risk for diabetes related complications [1]; therefore, finding ways to improve adherence to therapy is crucial. T1D affects more than 1 in 400 youth in the United States [2], making it one of the most common chronic childhood health conditions. The intensive treatment plan recommended for T1D requires a daily regimen of blood glucose checks, insulin administration, carbohydrate counting, and many other tasks (see Figure 1) that can become burdensome and challenging over time [3,4]. While the management of T1D is difficult at all ages, it can be particularly challenging during adolescence, when the interplay of physiologic and psychosocial changes can negatively affect glycemic control [5]. Maintaining good glycemic control can decrease the risk of complications such as retinopathy and nephropathy, but only 17% percent of adolescents with T1D meet American Diabetes Association targets for hemoglobin A1C [6], a surrogate for disease control. Additionally, while glycemic control is poorest during adolescence, it is better both before and after adolescence [7,8]. Adolescents with T1D are clearly a high-risk population; therefore, minimizing the challenges of diabetes management in this population is important. Recent reviews have examined various facilitators and barriers of adolescents’ adherence to treatment, such as parental involvement [9], peer influence [10], psychological adjustment [11], and access to care [12]. A potentially important and modifiable factor that has received less attention in the pediatric diabetes arena is patient-provider communication, and how this communication is linked to adherence to therapy.

Adolescents experience significant physiological and psychosocial changes during this developmental stage, including increased insulin resistance related to pubertal hormones, significant weight gain, higher insulin needs [13], and increasing independence from parents. Unfortunately, this newfound autonomy often results in problems with adherence and may further exacerbate poor glycemic control [14]. In addition, adolescents with chronic conditions may be more likely to engage in high-risk behaviors, such as drug and alcohol use, cigarette smoking, and unprotected sexual activity, than their peers [15], and these risky behaviors have the potential for greater adverse health outcomes in youth with chronic health conditions [16]. In their most recent practice guidelines, the American Diabetes Association (ADA) noted the difficulties in managing diabetes during this period and recommended that providers discuss potential stressors during visits [3]. Thus, understanding the important role of communication between patients, their caregivers, and providers, and addressing challenges between these groups may help adolescents improve adherence and achieve optimal glycemic control. We use the term “adherence” as it acknowledges the active role of patients in developing and following a treatment plan [17,18]. In this review, we synthesize the recent literature on patient-provider communication in pediatric populations, with a focus on the factors that are developmentally relevant, modifiable, and may be addressed by providers, including time management, technology, health numeracy, and provider burnout. We also note implications for practice, by offering recommendations to improve communication between patients, caregivers, and providers.

## 2. Importance and Function of Communication

Communication can be an effective tool to create a connection and share information between healthcare providers, patients, and family members. Verbal communication in the healthcare arena is defined as the act of exchanging information in order to identify the medical issue [19]. Non-verbal communication is the emotional tone of an interaction between people and includes the signals given off by how a person speaks, stands, looks, and acts when speaking with another person. The functions of communication affect health outcomes either directly or indirectly [20]. For example, when a provider verbally discusses key concerns and sympathizes with patients about their specific symptoms, there is a positive influence on health outcomes and emotional well-being (i.e., direct pathway), as well as increased trust and understanding between the patient and provider (i.e., indirect pathway) [20].

The patient-provider relationship is strongly related to adherence to treatment (i.e., following the recommended regimen), health outcomes, and patient and provider satisfaction in all medical conditions [18,19], but it may be especially important for chronic pediatric conditions. Pediatric providers need to communicate effectively, not only with their patients, but also with their patients’ caregivers, which may explain why, in a meta-analysis, the association between physician communication and patient adherence was found to be stronger for pediatricians (*r* = 0.25) than adult physicians (*r* = 0.18) [18]. By communicating effectively with the patient’s family members, providers can obtain caregivers’ insight and knowledge of the patient’s behavior and preferences, which has the potential to increase adherence to treatment.

Successful communication between healthcare providers and patients’ caregivers also improves satisfaction with care [21]. For example, in a study of youth with chronic conditions (e.g., asthma, diabetes mellitus, or sickle-cell disease) using videotapes of clinic visits, investigators found a significant association between clinic visit satisfaction (based on parent report) and observed friendliness of physicians towards parents [22]. Similarly, in a national survey of parents of youth with diabetes (SEARCH study), 43% of parents reported that communication was a barrier to care, and 48% of parents reported that getting information (defined by having questions discussed/answered) was a barrier to care [23]. These findings are concerning in that patients’ caregivers report feeling unable to communicate with providers to allow their questions and concerns to be addressed and answered. But when providers communicate effectively, the result is enhanced transmission and retrieval of important clinical information (e.g., better description of symptoms) and improved understanding of the treatment plan [18], all of which have the potential to increase adherence and improve health outcomes.

Communication with patients is particularly important in pediatric diabetes, given the complexity of the recommended treatment plan and frequency of clinic visits [3]. However, many adolescents with diabetes perceive clinic visits as stressful and negative, describing concerns about confrontation regarding poor glycemic control [24]. Given this negative connotation around provider visits, adolescents are not always willing to exchange important information with providers. A qualitative study of adolescents’ experiences of communicating with health professionals highlighted several barriers to good communication between patients and providers [25]. Specifically, adolescents with chronic illness described a reluctance to discuss personal or sensitive issues (especially when parents or other health professionals were in the room) and to ask questions that revealed problems with adherence. Further, adolescents reported feeling as though they were unable to exchange information when the provider had poor communication skills or when the provider focused on the adolescent’s health condition without asking about other aspects of the adolescent’s life. Similarly, adolescents say that they value health care professionals who take time to offer emotional support [24]. For example, deWit and colleagues demonstrated that asking adolescents about quality of life during clinic visits improved psychosocial well-being and diabetes-related family conflict, and adolescents reported greater satisfaction with care [26]. Providers can also improve outcomes by supporting patient autonomy in developing a treatment plan [20]. Thus, it is important for providers to acknowledge the needs of adolescents and treat them as a critical member of the decision-making process to achieve optimal adherence to the complex treatment regimen.

## 3. Essential Factors to Improve Communication

Adolescence is a high-risk time for patients with type 1 diabetes, as it is associated with poor glycemic control [6] and problems with adherence [5] as youth take over more responsibility for management. In the following sections, we identify potential factors to improve communication and note implications for practice, by offering recommendations to help providers communicate effectively with adolescents and their caregivers (see Table 1). Since a discussion of all of the factors that may influence communication is beyond the scope of this paper, we focused on time management, technology, health numeracy, and provider burnout as potentially modifiable, provider-led factors that may improve adherence in pediatric diabetes.

## 4. Time Management in the Era of the Electronic Medical Record

Successful communication requires time for providers to communicate with patients and their caregivers; however, with increased use of technology, providers are spending less time in direct patient care, and more time entering data into an electronic system of care [27,28]. This is especially concerning in the context of adolescent visits due to the amount of information providers need to discuss during visits, and the inherent differences in treating adolescents (compared to other developmental stages). The ADA identifies several issues specific to adolescence that need to be addressed by providers, including changes in insulin requirements due to puberty, concerns about self-image, risk of behavioral problems such as depression, transition to self-management, and transition to an adult diabetes provider [29]. In addition, the ADA recommends that providers spend part of each clinic visit alone with adolescents to address these issues [3]. Recent data from the preventive medicine field suggests that in visits that were “partially confidential,” in which the adolescent was allowed private discussion time with the provider, there was a statistically significant increase in the number of issues discussed during the visit [30]. The authors of this study suggest that a “split-visit model,” in which caregivers participate for a portion of the visit and allow adolescents time alone with their providers, is the most fruitful and allows the most discussion [30], particularly around risk behaviors such as drug and alcohol use, cigarette smoking, and unprotected sexual activity [15]. The unique challenges in caring for adolescents with T1D make time management even more critical to maximize time with patients and their caregivers.

With the mandate to use electronic medical records (EMR), it is especially important for providers to manage their time and maintain good communication throughout the clinic visit with each patient. In a review by Shachak and Reis [31], both positive and negative effects of the EMR on patient-provider communication emerged. Specifically, the authors noted a positive effect on information exchange, but a negative effect on “patient centeredness”. The ways in which physicians used the computer during visits influenced their ability to communicate with their patients; while some (the “informational-ignoring physicians”) lost rapport with patients because they relied too heavily on the computer at the expense of facing the patient and making regular eye contact, others (the “interpersonal style” physicians) focused more on the patient, spoke to patients without typing, and did not use the computer at the beginning of the encounter. Although this study was not specific to pediatric patients, the findings are relevant to pediatric providers who are communicating with patients and their adult caregivers.

Exploring the specifics of the patient encounter enriches our understanding of how to keep office visits patient centered. A recent study of adult patients examined videotaped patient-provider visits, in which providers used a computer and the electronic health record during the visit. In this sample, patient-centered communication decreased when providers spent more time looking at their computers and when there was increased conversational dead space (defined as time when neither the patient nor the provider was talking) [32]. These findings suggest that providers should learn strategies to maximize patient centeredness and avoid spending the entire patient encounter entering information into the medical record [31,32]. For example, researchers recommend that providers learn “blind typing” (i.e., typing while looking at the patient), start with patient concerns, learn to find patient resources rapidly online, separate data entry from the encounter, point to the screen and highlight data to show the patient, and look at patients during the clinic appointment (e.g., push the monitor away when listening).

Given the time constraints on pediatric providers during routine clinic visits, new time-saving strategies are being developed and tested. For example, conducting screenings prior to the actual patient-physician encounter may streamline patient visits. One study demonstrated that a computerized assessment of health behaviors completed by adolescents in the waiting room (before the visit) allowed physicians to focus on areas of concern with their patients, resulting in positive effects on adherence to recommendations for nutrition and physical activity level [33]. Pediatric diabetes providers could broaden the scope of such a tool to include other relevant information that could then facilitate a discussion during the patient-physician encounter, such as asking about quality of life [26]. For youth who are uncomfortable speaking with providers, creating a list of questions or topics to discuss prior to the visit may also be helpful [24]. Scheduling time for patients and caregivers to meet with a certified diabetes educator may also allow for greater discussion of barriers to adherence than can be addressed during a visit with the physician or nurse practitioner.

Providers have recently explored the use of group visits or shared medical appointments (appointments with multiple patients seen together) as a way to avoid repeating the same information to individual patients and increase direct point of care between the provider and patient [34]. With conditions such as T1D, many families are experiencing similar challenges, and a group setting may allow adolescents and caregivers to share strategies to overcome those challenges [35]. Thus, group appointments may have the indirect benefit of forming a support system for families of youth with T1D. Shared appointments may also present an opportunity for providers to model effective communication around diabetes management. For example, providers can teach and encourage caregivers to review logbooks with their adolescent and talk about “high” and “low” numbers calmly. Given the multiple demands on their time, providers should work to balance their time between direct and indirect patient care (e.g., note entry) and use available resources when possible to maximize time with adolescent patients for discussion of barriers and facilitators to self-management.

## 5. Technology as a Communication Tool

Providers are increasingly using technology as a tool to increase communication and help adolescents manage their diabetes. Health information technologies, such as automated messages and patient portals, have the potential to facilitate patient-provider communication, and may even improve delivery of care [36]. Adolescents are more likely than adults to use internet-based technologies, due to the flexibility, easy accessibility, and innovative strategies technology allows [37]; therefore, interventions that employ technology may serve as an excellent portal for patient-provider communication. Mobile phone-based options may be especially appealing to adolescents, since 88% of adolescents 13–17 have access to a mobile phone [38]. The use of technology is also an emerging option for patients that live far from medical centers or may not be able to come into diabetes clinic with concerns. For example, Carroll and colleagues [39] tested an intervention that used a cell phone device (Glucophone^TM^) to automatically deliver adolescent patients’ blood glucose values to providers’ computers. This data allowed providers to evaluate and offer therapeutic consultations in real time and reduced the worry and concerns of parents. More recently, researchers tested the effects of a mobile app to improve self-management in adolescents with T1D. In a randomized clinical trial comparing adolescents who received the app to usual care, no differences were found in any of the outcomes, including frequency of blood glucose monitoring, glycemic control, or quality of life, and the study authors hypothesized that sharing blood glucose data with providers may have increased the effect of the intervention [40]. Similarly, the feasibility and acceptability of text messaging interventions for youth with T1D has been demonstrated, but the effects on diabetes-related outcomes have been inconsistent [41]. In short, it may be useful for providers of adolescent patients to include an online or technology-based option in their diabetes treatment plans. However, in order to maintain gains in adherence, technology must be integrated with other components of interpersonal communication [42].

## 6. Health Numeracy

Health numeracy is another important component of health communication in pediatric diabetes, where frequent interpretation of numbers is necessary. There are many skills involved in health numeracy, including the ability to compute basic and multistep math functions and interpret measurements, graphs, and time [43]. In pediatric diabetes, patients (and their caregivers) must be able to identify abnormal blood sugars, calculate correction doses of insulin based on blood sugars that are out of the target range, count carbohydrates and match their meal intake with insulin, and understand general trends of their blood sugars to adjust insulin doses as necessary. Assessing the health numeracy of adolescent patients and their caregivers has potential to improve adherence, given the complexity of the diabetes regimen in the setting of puberty (and changing insulin requirements), school, work, and social changes, and the increased independence of diabetes management.

In a recent study, lower health numeracy among adolescents with diabetes was related to caregivers taking greater responsibility for diabetes care and lower diabetes problem-solving skills in adolescents [44]. These findings support the need for providers to gauge patients’ and caregivers’ numeracy levels in order to provide appropriate information regarding nutrition, insulin adjustments, and glucose monitoring [45]. For example, if a patient or his/her caregivers cannot read food labels or interpret serving sizes (and therefore are unsure of how many carbohydrates they are eating), the treatment plan is doomed to fail [46]. Providers may find it valuable to provide a hypothetical situation (e.g., asking patients to calculate a mealtime insulin dose using a hypothetical blood sugar and number of carbohydrates eaten) to determine the health numeracy of the patient and/or caregiver, ensure that patients are able to calculate insulin doses correctly, and determine readiness for advanced technologies such as insulin pumps and continuous glucose monitoring systems. In addition, providers may consider using simpler ratios (10 g of carbohydrates: 1 unit of insulin vs. 7:1) when making treatment recommendations to help patients that are experiencing numeracy problems. Pediatric providers are crucial stakeholders in the health of adolescents with diabetes [47]; therefore, it is essential that they are able to successfully communicate the specific components of the treatment plan by taking into account patients’ health numeracy.

## 7. Provider Burnout

Provider burnout may also contribute to poor communication among patients and providers. In recent years, the requirements and obligations of providers have increased; many providers are expected to see patients while meeting external obligations, such as conducting scientific research, mentoring medical students, and engaging in continuing education or career development programs [48]. Providers report stress related to increased workload, reduced control over work environment, and frustration with patients’ poor adherence to treatment recommendations [49]. Studies show that 28% of health care providers show clinically significant distress levels [50], and these rates may be increasing. For example, Shenafelt et al. found that physicians’ reports of burnout/satisfaction with work-life balance has increased from 45.5% in 2011 to 54.4% in 2014 (*p* < 0.001) [51]. Provider burnout is associated with impairment in patient interaction and communication, deteriorating productivity, and decreased empathy [49,52].

Taking care of adolescent patients with diabetes may be particularly stressful, given the frequency of problems with adherence and challenges meeting treatment goals in this age group. Therefore, stress-reduction programs are needed to alleviate provider burnout that may have a negative impact on communication. For example, mindfulness programs, which focus on “the ability to pay attention in a particular way; on purpose, in the present moment, and nonjudgmentally” [53] have proven effective for decreasing physician burnout and increasing personal accomplishment and empathy [54]. Further, medical centers have made efforts to address burnout by providing resources for providers. For example, a pilot program at Stanford University allowed faculty to trade time spent on committees and other administrative duties for in-home support, such as meal delivery and cleaning services, or work-related support, such as grant writing assistance. This program increased the feeling of being supported, especially among female faculty [55]. In order to increase positive health outcomes among both themselves and their patients, providers should monitor stress levels and seek out resources within the workplace or community for reducing and managing stress. By preventing and addressing burnout, providers may be more effective in their interactions with patients [52].

## 8. Implications for Practice

Communication between the patient, caregiver, and provider is an essential component of promoting adherence in pediatric diabetes. Fortunately, research supports the notion that communication skills are a modifiable factor [56]; a meta-analysis of communication training programs for physicians’ demonstrated increases in collaborative communication, empathy, and improved attitudes toward psychosocial issues [56]. For example, a recent study used standardized patients to enact phone call counseling scenarios to improve physician (pediatrician or family physician) communication. Physicians were asked to call the “mother” (standardized patient) of a newborn who had specific newborn screening results that needed to be reviewed. After the baseline counseling phone call, the intervention group received a report card providing feedback and outlining various aspects of their communication. Both the intervention and control groups then performed another phone counseling session with a standardized patient. The authors found that the intervention group improved in certain areas of all four of the quality indicators studied (“assessment of understanding,” “organizing behaviors,” “precautionary empathy,” and “jargon” use) [57]. These findings highlight specific ways that training could improve providers’ communication in pediatric diabetes by providing autonomy support to patients and taking a more collaborative approach [58].

In addition, training in cultural competency has the potential to improve providers’ communication skills. It is well established that a better understanding of patients’ cultural beliefs, values, and traditions also improves communication and may increase disclosure of personal health information [59]. However, a recent review by Twomey [60] highlighted the lack of culturally competent communication among pediatric healthcare providers and their patients, concluding that many providers lack the training required to provide optimal care among culturally diverse populations. To address these deficits, many healthcare systems are gradually integrating training programs related to cultural sensitivity that helps providers to be more effective in communicating with ethnic minorities. In a systematic review of 34 studies on this topic, the authors determined that there is no one specific way to train providers about culture; however, there is substantial evidence that cultural competency training improves clinicians’ knowledge and impacts the attitude and skills of healthcare providers [61].

Finally, the recent changes to the Medical College Admission Test (which is required for admission to medical school) to include a section on social and behavioral sciences is noteworthy in regards to provider communication [62]. The Association of American Medical Colleges hopes that the increased focus on social and health care issues will result in more well-rounded physicians. Changing the way providers are trained and requiring that providers understand the behavioral and socio-cultural issues of their patients, signals an important shift toward acknowledging the importance of provider communication. Similarly, the inclusion of patient-centered communication in the ADA’s 2015 recommendation for care reflects this shift [63].

Effective communication between providers, patients, and caregivers is essential to mitigate the many challenges facing pediatric patients with T1D and has the potential to improve adherence in patients with T1D. In this review, we identified several potentially modifiable factors that can impede successful communication and offered recommendations for providers to address these barriers (see Table 1). More research is needed to determine the most effective, specific communication strategies for providers who care for adolescents with T1D. Given that adolescents with T1D are a high-risk sample, even small improvements in patient-provider communication have the potential to improve adherence and other outcomes.

## Figures and Tables

**Table 1:** Essential Factors for Improving Communication.

Factor	Recommendations
Time	▪ Pre-visit computerized assessments ▪ Use EMR Effectively ▪ Group visits
Technology	▪ Text messaging as communication tool ▪ Integrate technology with interpersonal communication skills
Health Numeracy	▪ Practice hypothetical situations during clinic visits ▪ Simplify treatment recommendations based on patients’ numeracy level
Provider Burnout	▪ Practice stress reduction strategies (e.g., mindfulness training) ▪ Seek out workplace resources
